# The role of autophagy in the treatment of BRAF mutant colorectal carcinomas differs based on microsatellite instability status

**DOI:** 10.1371/journal.pone.0207227

**Published:** 2018-11-14

**Authors:** Evangelos Koustas, Athanasios G. Papavassiliou, Michalis V. Karamouzis

**Affiliations:** 1 Molecular Oncology Unit, Department of Biological Chemistry, Medical School, National and Kapodistrian University of Athens, Athens, Greece; 2 First Department of Internal Medicine, ‘Laiko’ General Hospital, Medical School, National and Kapodistrian University of Athens, Athens, Greece; Univerzitet u Beogradu, SERBIA

## Abstract

Autophagy has been identified as a catabolic mechanism in cells but its’ role in cancer remains controversial. Autophagy has been characterized either as tumor suppressor or inducer mechanism in many tumor types. Monoclonal antibodies against EGFR (cetuximab and panitumumab) represent a major step in the treatment of mCRC. Several studies propose that cetuximab and panitumumab trigger autophagy which reveals a potential resistance mechanism to these agents. The last years immunotherapy appears to be a novel promising strategy for the treatment of patients with solid tumors, including colorectal cancer. Checkpoint inhibitors, such as anti-PD1 (nivolumab and pembrolizumab) and anti-CTLA-4 (ipilimumab) antibodies have already been developed and applied in mCRC patients with MSI-H phenotype. The association between mtBRAF and autophagy or MSI status has already been characterized. In our study, we identify the autophagy initiation through anti-EGFR monoclonal antibodies and checkpoint inhibitors in colorectal carcinoma cell lines according to microsatellite status. The combination of autophagy inhibition, anti-EGFR antibodies and checkpoint inhibitors as well as autophagy targeting, MEK inhibition and anti-EGFR antibodies or checkpoint inhibitors appears to be the best treatment approach for microsatellite instability high and stable colorectal cancer cell lines, respectively. Both combinatorial approaches reduce cell viability through the induction of apoptotic cell death. The findings of this study point out the importance of different approach for the treatment of BRAF mutant metastatic colorectal cancers based on their microsatelite instability phenotype.

## Introduction

Colorectal cancer (CRC) is one of the most commonly diagnosed malignancy which leading to cancer-related deaths in the world. CRC r is expected to increase more than 50% by 2030 [1]. Some patients are diagnosed with metastases, while 20% of CRC patients will eventually develop metastases, thus, emphasizing the importance of novel effective treatment options [2,3].

The expression of epidermal growth factor receptor (EGFR) has been identified as key molecule in several human cancers, including mCRC [4]. During the last decade, anti-EGFR monoclonal antibodies (mAbs), such as Cetuximab and panitumumab, were shown to add significant survival benefit in combination with traditional chemotherapy [5]. Unfortunately, acquired resistance eventually develops against anti-EGFR mAbs in mCRC patients. Mutations in proto-oncogenes, such as RAS or BRAF, have been identified as an important resistance mechanism of anti-EGFR mAbs [6,7]. BRAF mutations, especially BRAF^V600E^, in patients treated with anti-EGFR mAbs seem to be predictive of treatment unresponsiveness [8]. Moreover, clinical trials suggest that anti-EGFR mAbs probably do not enhance the efficacy of chemotherapy in tumors with BRAF^V600E^ mutation [9,10].

Many studies have shown that EGFR and BRAF regulate the cytoprotective mechanism of autophagy, a self-digesting process in cells [11,12]. The mechanism of autophagy has been proposed as a key element to improve the efficacy of anti-EGFR mAbs in several tumors, including mCRC [10]. Therefore, autophagy is expected to become a new treatment target for different cancers [13]. The identification of autophagy as a cytoprotective mechanism against several anticancer agents has potentiated to use autophagic inhibitors as a new form of cancer therapy treatment. Targeting autophagy represents a promising approach to overcome the resistance against cancer therapy. [14,15]. The role of autophagy as cytoprotective mechanism needs further investigation, while the association of autophagy with carcinogenesis may depends on stage and size of tumor [16].

Furthermore, except the regulation of autophagy, mt BRAF seems to play a crucial role also in sporadic high microsatellite instability (MSI-H) tumors. It has already been identified the association between of MSI-H status and mtBRAF in CRC tumors through CpG island methylator phenotype (CIMP) [17]. In addition, the presence of MSI-H phenotype is observed in about 15–20% of sporadic CRC and it has been associated with a less aggressive phenotype, and a better prognosis compared to patients with microsatellite stable (MSS) phenotype. [18,19]. Moreover, MSI-H tumors are characterized from a high number of specific neo-antigens which presented on MHC and recognized by T cells [20]. These neo-antigens may explain, in part, the high amount of TILs (tumor-infiltrating lymphocytes) in MSI-H compared to MSS CRC tumors [21].

Tumors with MSI-H phenotype represent the initial subset of CRC where immunotherapies have seen successful [22]. Many years of research have given some encouraging results in the immunotherapy approach of CRC, namely PD-1 inhibition in tumors with MSI-H phenotype. In different cancers, the protein levels of PD-L1 (the ligand for PD1) has been found highly expressed [23,24]. The last few years mAbs against PD-1 and its ligand PD-L1 have been developed and increase the effectiveness of immune system against many cancers types [23,24]. Furthermore, many clinical studies evaluate therapy with anti-PD1 (such as nivolumab and pembrolizumab) alone or with anti-CTLA4 (such as ipilimumab) mAbs. This combination of checkpoint inhibitors seems to be more effective in a variety of cancers [25]. While, there is a specific role for PD-1 inhibition in MSI-H CRC, for MSS CRC, alternative approaches will be required. Agents against MEK and PD-L1 in combinatorial schemes are being rigorously tested in MSS tumors and has shown synergistic antitumor activity [26,27].

In the present study, we examined the role of autophagy (A) as a cytoprotective mechanism against anti-EGFR mAbs (E) and checkpoint inhibitors (I) in CRC cell lines. In our experiments, we observed that anti-cancer agents, E and I, appear to trigger autophagy in CRC cell lines. Overall, these results indicate that the triple scheme of A inhibition combined with E and I antibodies, represents a promising treatment approach in mCRC with MSI-H phenotype. Moreover, for MSS cell lines the best approach is the combination of A and MEK inhibitors with anti-EGFR or checkpoint inhibitors This study proposes a putative combinatorial treatment using a specific inhibitor against autophagy, 3-MA or HCQ, which triggers apoptotic cell death. The association of mtBRAF with autophagy and MSI-H phenotype makes these combinatorial approaches a promising therapeutic approach that needs further testing.

## Materials and methods

### Inhibitors and drugs

BRAF/MEK/ERK signaling pathways were inhibited using specific MEK kinase inhibitors PD 0325901 #PZ0162 SIGMA-ALDRICH. Autophagy inhibitors: 3-Methyladeninee (3-MA), #13242 CAYMANCHEMICAL COMPANY, Hydroxychloroquine #H0915 SIGMA-ALDRICH. Anti-EGFR MoAbs Erbitux (Cetuximab) Merck KGaA and Vectibix (Panitumumab) Amgen Europe B.V, checkpoint inhibitors; Nivolumab (a human programmed death receptor-1 (PD-1) blocking antibody) (Bristol Mayer Squibb), Pembrolizumab (a human programmed death receptor-1 (PD-1) blocking antibody) (Merck KGaA), Ipilimumab (Bristol Mayer Squibb).

### Cell lines

RKO (ATCC CRL-2577), Colo-205 (ATCC CCL-222) and HCT116 (ATCC CCL-247) human colon adenocarcinoma and Caco-2 (ATCC HTB-37) colon intermediate adenoma cell lines were obtained from American Type Culture Collection (ATCC). All cell lines used in this study were grown in D-MEM medium supplemented with 10% fetal bovine serum (FBS), L-glutamine, vitamins, penicillin, and streptomycin antibiotics and amino acids (all from Invitrogen). Cells were maintained at 37°C in a humidified incubator containing 5% CO2. The experiments were done with the approval of the Ethics Committee of our University.

### Cell viability assay

At the end of treatment and incubation time, CRC cell lines were incubated for 4 h with 0.8 mg/ml of MTT, dissolved in serum free medium. Washing with PBS (1 ml) was followed by the addition of DMSO (1 ml) and gentle shaking for 10 min to achieve the complete dissolution. In every plate of 96-well plate 200 μl of the resulting solutions were added and absorbance was measure at 560 nm using the microplate spectrophotometer system (Spectra max190-Molecular Devices). Results are presented as percentage of the control values.

### Western blotting

As described in detail previously [12], after incubation time, RIPA buffer is used for the preparation of whole cell lysates. The protein concentration was determined using the Bradford method (Bio-Rad, 5000006). A total of 25 μg of protein was resolved on SDS-PAGE and transferred to nitrocellulose membrane (Whatman, Scheicher & Schuell, Dassel, Germany). Membranes were incubated with the primaries antibodies overnight at 4°C. After incubation time, membranes are washed with TBS-T and then incubated with the appropriate secondary antibody, for 1 h at 24°C. Antibodies were used against: pEGFR #3777, pERK #2211, LC3B(D11) #3868, SQSTM1/p62 #8025, cleaved caspase-3 #9661, PARP-1 #9542, PD-L1 #13684 from Cell Signaling (Danvers, MA, USA) and Actin (sc-8035) from Santa Cruz (Biotechnology, Inc. 2145 Delaware Avenue Santa Cruz, CA 95060 USA). Signal of antibody was identified with the enhanced chemiluminescence and specific detection system (Amersham Biosciences, Uppsala, Sweden) after exposure to FUJI MEDICAL X-RAY FILM. The amount of protein levels were measured using specific software (Image-Quant software- Amersham Biosciences). The normalization of protein levels is against actin. The experiments represent three independently experiments and standard deviation is presented.

The protein band intensities were measured by Imagej and normalized to each corresponding loading control actin.

### Three dimension culture

As described in detail previously [12], the CRC cell lines were grown in 24-well plates on 25% Matrigel (BD Bioscience) in 37°C for 15 minutes order to form a gel line of 1 mm thickness. 600 μl of total volume (2×104 cells mixed 1:1 with 4% Matrigel) was covered the bottom later. Every two days D-MEM containing 2% of matrigel was replaced in each plate. The cells were left to grow for 13–15 days to allow development tumors, after which treatment were applied for indicated incubation times. Photographs of the three-dimensional cultures were taken using a Olympus FV1000 confocal microscope with an Olympus digital camera. The nuclei were stained with Dapi No. 33342. The cleaved caspase-3 was detected with cleaved caspase-3 specific antibody.

### Statistical analysis

The results are representative out from at least three independent experiments and expressed as mean values ± SD (standard deviation). The results were evaluated by TTEST. Statistical significance was inferred when *P<0*.*05*.

## Results

### Steady levels in basic colon adenocarcinoma cell lines

**Fig 1:**
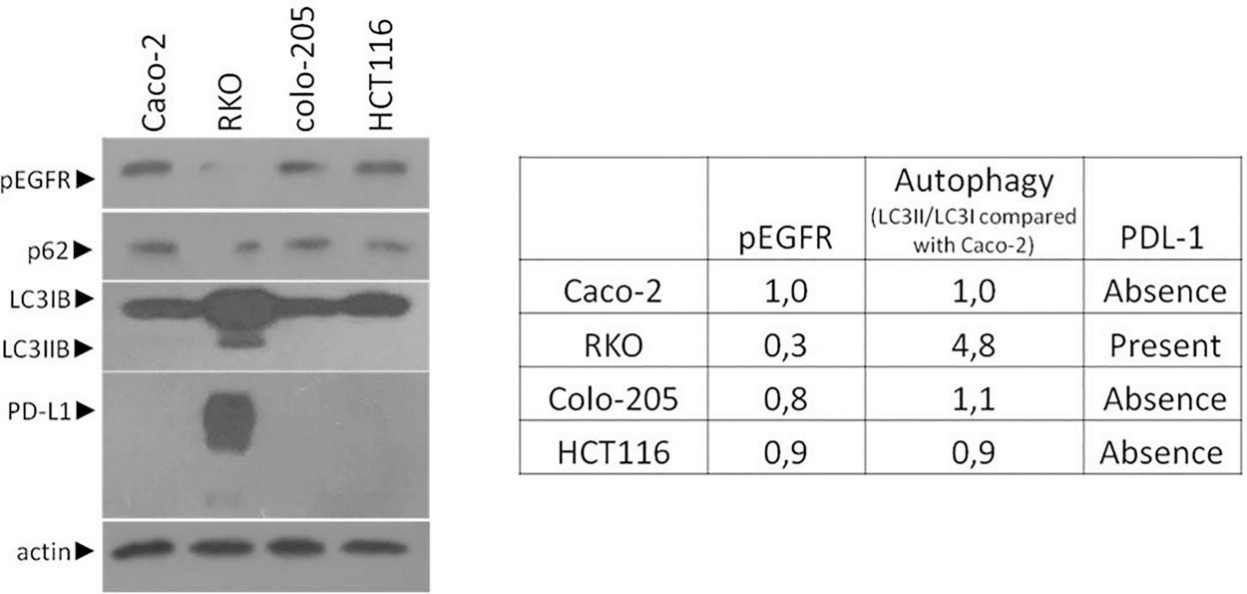
Steady-state levels of colon cancer cell lines. Using Western blot assay steady protein levels of pEGFR, autophagy markers LC3B, and PD-L1 analyzed in basic colon adenocarcinoma cell lines (Caco-2, RKO, colo-205 and HCT116). As a control cell, line Caco-2 is used as an intermediate adenoma cell line. The quantification of LC3 reflects the ratio of LC3II/LC3I in comparison with Caco-2 in each sample separately. Each cell line was compared with Caco-2 for the protein levels of pEGFR, autophagy levels and the presence or absence of PD-L1 protein levels.

Different colon adenocarcinoma cell lines were examined regarding their autophagic properties (through the ratio of LC3II/LC3I), the protein levels of pEGFR and PD-L1 using western blot analysis. In detail, the protein levels of pEGFR and the ratio of LC3II/LC3I were found decreased and elevated, respectively in RKO and colo-205 adenocarcinoma cell line as compared to Caco-2. As an additional confirmation of autophagy, CRC cell lines were stained with MDC in a high percentage of Phalloidin stained cells (S1 Fig). HCT116 cell line (mtRAS and MSI-H phenotype) was tested for the same properties. The level of basic autophagy and increasing levels of EGFR was detected. Furthermore, PD-L1 is strongly expressed in RKO cell line **(Fig 1).** Furthermore, RKO and colo-205 showed enhanced autophagic properties and low levels of pEGFR. Notably, RKO exhibited properties of MSI-H phenotype. These results propose a putative relation between autophagy and MSI-H cell program. Mutation and microsatellite status of CRC cell lines of this study are presented in S1 Table.

### Autophagy inhibition sensitizes the cells in anti-EGFR mAbs and checkpoint inhibitors

Over-expression of PD-L1 on tumor cells has been found to impair antitumor immunity. RKO with the highest PD-L1 expression was selected and defined as the target CRC cell line to optimize the experimental efficiency. Since BRAF^V600E^ and MSI-H RKO cell line presented remarkable autophagic properties after treatment with anti-EGFR mAbs (Cetuximab, panitumumab—E), checkpoint inhibitors (nivolumab, pembrolizumab, ipilimumab—I) and (E+I). Moreover, the efficacy of treatments combined with an autophagy inhibitor was also tested in this cell line. Using the MTT-viability assay, the response of RKO cell line were measured after treatments with 1μΜ of E [Cetuximab (C) or panitumumab (P)], 0,5 μΜ I [(pembrolizumab (PE), nivolumab (NI), ipilimumab (IPI)] and 5mM autophagy inhibitor (A) 3-Methyadenine (3-MA). The entire combinatorial scheme and the effect on cell viability after 72 hours are presented in Table 1.

**Table 1:** The combination of E, I and A and the and the main effect on RKO CRC cell line.

RKO	treatment	viabillity	autophagy	apoptosis	RKO	treatment	viabillity	autophagy	apoptosis
E	C	NC	PR	NC	A	HCQ	+	IN	NC
	P	NC	PR	NC	A+E	HCQ+C	+	IN	NC
I	NI	NC	PR	NC		HCQ+P	+	IN	NC
	PE	NC	PR	NC	A+I	HCQ+NI	+	IN	NC
	IPI	NC	PR	NC		HCQ+PE	+	IN	NC
E+I	C+NI	NC	PR	NC		HCQ+IPI	+	IN	NC
	C+PE	NC	PR	NC	A+E+I	HCQ+C+NI	++	IN	PR
	C+IPI	NC	PR	NC		HCQ+C+PE	++	IN	PR
	P+NI	NC	PR	NC		HCQ+C+IPI	++	IN	PR
	P+PE	NC	PR	NC		HCQ+P+NI	++	IN	PR
	P+IPI	NC	PR	NC		HCQ+P+PE	++	IN	PR
A	3-MA	+	IN	NC		HCQ+P+IPI	++	IN	PR
A+E	3-MA+C	+	IN	NC					
	3-MA+P	+	IN	NC					
A+I	3-MA+NI	+	IN	NC					
	3-MA+PE	+	IN	NC					
	3-MA+IPI	+	IN	NC					
A+E+I	3-MA+C+NI	++	IN	PR					
	3-MA+C+PE	++	IN	PR					
	3-MA+C+IPI	++	IN	PR					
	3-MA+P+NI	++	IN	PR					
	3-MA+P+PE	++	IN	PR					
	3-MA+P+IPI	++	IN	PR					

NC: not changed; IN: Inhibition; PR: present; +: <20% protein vs control; ++: <40%

**Fig 2:**
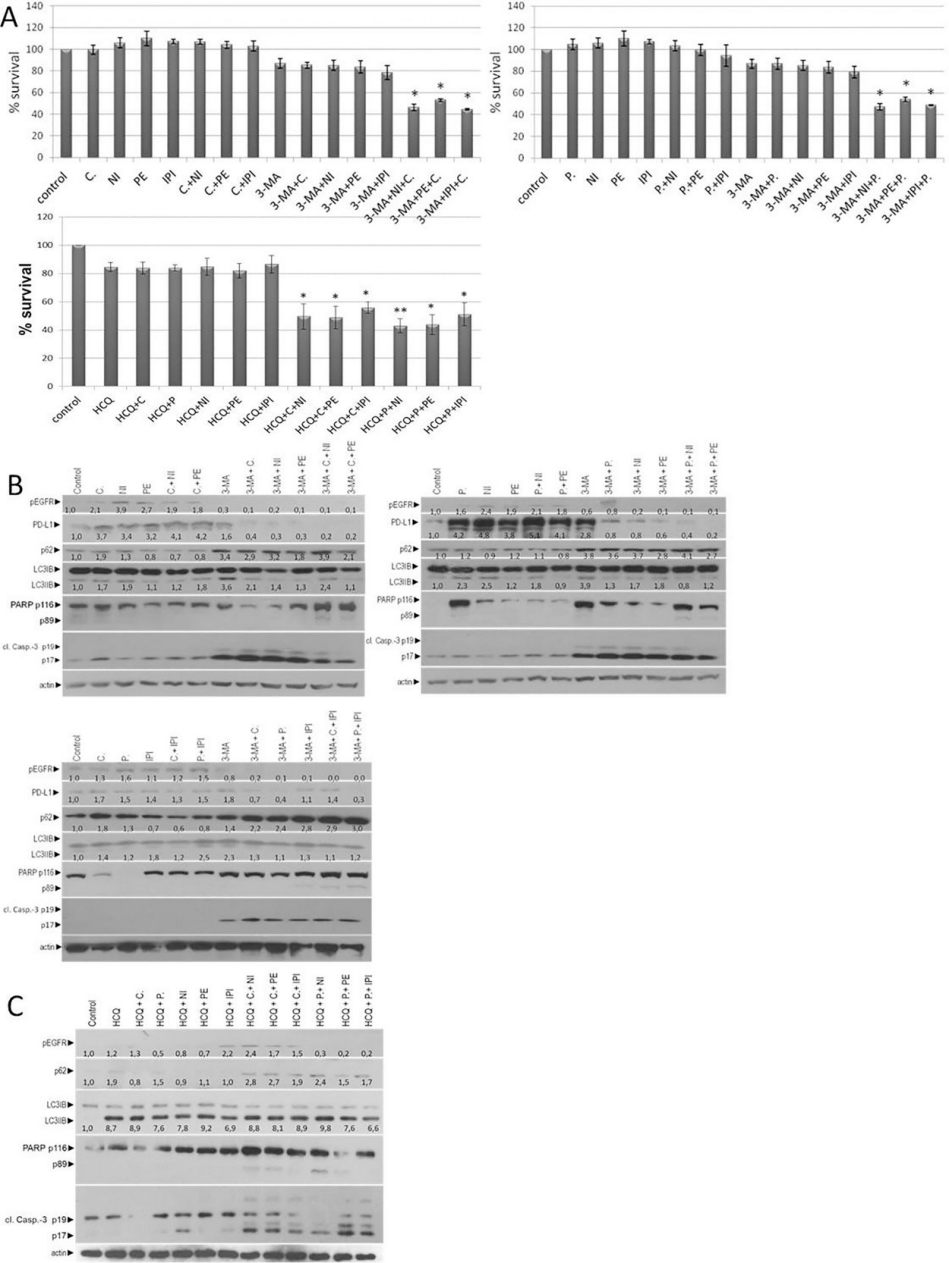
Autophagy inhibition enhances combined immunotherapy and anti-EGFR treatment in MSI-H colon cancer cell line. (**A**) Cell viability of the mutant BRAF^V600E^ colon cancer cell lines RKO after 72 hours treatments with 1μM anti-EGFR mAbs Cetuximab (C) or panitumumab (P), 0,5 μM checkpoint inhibitors nivolumab (N), Pembrolizumab (PE) or ipilimumab (IPI), 5mM of the autophagic inhibitor 3-MA (A) or 20μΜ of Hydroxychloroquine (HCQ) and in combination with a constant dose of E (C or P) and /or I (E+I, A+E, A+I and A+E+I). (**B**) Western blot analysis after 24 hours exposure of cells alone or in combination with a constant dose of E, I E+I, A+E, A+I and A+E+I. The protein levels of apoptotic cell death were identified by antibody against PARP and cl. caspase-3. The protein levels of p-EGFR, PD-1, LC3 and p62 are also presented. The quantification of LC3 reflects the ratio of LC3II/LC3I in comparison with control in each sample separately. Protein levels were normalized against actin. (**C**) Western blot analysis after treatment of RKO for 24 hours with 0,5 μΜ I, 1μΜ of E and 20μΜ of autophagy inhibitor (A) Hydroxychloroquine (HCQ), alone or in combination of A, A+E, A+I, A+E+I. The detection of p-EGFR, LCE3, p62, PARP and cl. Caspase 3 is tested by specific antibodies against each protein. The quantification of LC3 reflects the ratio of LC3II/LC3I in comparison with control in each sample separately. Protein levels were normalized against actin.

Due to inhibition of autophagy, cell viability was reduced up to 13,2% (A+P), 14,8% (A+C), 14,9% (A+NI), 16,4% (A+PE) and 21% (A+IPI) in 72 hours. Further inhibition (25% more) of cell growth was observed in triple inhibition of A+E+I. The total cell viability reduction in comparison with control was around 50–55% after 72 hours **(Fig 2A).** Furthermore, RKO CRC cell lines treated also with 20μΜ of a second autophagic flux inhibitor hydroxychloroquine (HCQ). All combinatorial schemes are shown in Table 1. Triple inhibition of A+E+I, using HCQ, reduce the cell viability in average of 42–55% after 72 hours **(Fig 2A).** These data suggest that inhibition of autophagy sensitized and reduced the number of MSI-H RKO cells in anti-EGFR mAbs (E) and checkpoint inhibitors (I) treatment.

### Triple inhibition of (A), (E) and (I) induces apoptosis in BRAF^V600E^/MSI-H colorectal cancer cell line

As a next step, we investigated the way of reduction in cell viability of colorectal cancer cell line RKO. RKO cell line was exposed alone to 1μΜ of E (C or P), 0,5 μΜ I (PE, NI, IPI) and 5mM autophagy inhibitor (A) 3-Methyadenine (3-MA). The entire combinatorial scheme and the main effect after 24 hours are presented in Table 1 Treatment with C, P, NI, PE, IPI, C+NI, C+PE, C+IPI, P+NI, P+PE, P+IPI induces pEGFR (1.1 to 7.7 folds), PD-L1 (1.2 to 4.7 folds). The increasing levels of autophagy were identified through the increasing levels of LC3II/I ratio after treatment with C, P, NI, PE, IPI, C+NI, C+PE, C+IPI, P+NI, P+PE, P+IPI in comparison with control (1.2 to 5.8 folds) and reduction of p62 after treatment with NI, PE, IPI, C+NI, C+PE, C+IPI, P+NI, P+PE and P+IPI (0.4 to 0.9 folds) **(Fig 2B).** Due to inhibition of autophagy (A+E, A+I and A+E+I) the levels of pEGFR decreased around 0.7 to 0.0 as compared with untreated cells. Furthermore, the protein levels of PD-L1 in A+E, A+NI, A+PE, A+E+NI, A+E+PE and A+P+IPI were decreased (0.1 to 0.9 folds). Interestingly, the increasing levels of p62 and LC3II/I ratio were found upon longer 3-MA treatment in RKO cells, confirming the inhibition of autophagy and accumulation of LC3 in early vacuoles **(Fig 2B)**. Moreover, treatment with 5mM 3-MA resulted in apoptotic cell death in the double and triple combinatorial scheme (A+E, A+I and A+E+I). The detection of PARP-1 and caspase-3 cleavage by western blot analysis, identified the apoptotic mechanism which is involved in the reduction of RKO after inhibition of autophagy **(Fig 2B).** Taking all the above into consideration, it can be proposed that autophagy has a cytoprotective role in colorectal cancer cell line RKO, since inhibition of autophagy with 3-Methyladenine was remarkably correlated with decrease in cell viability and appearance of apoptosis. In addition, inhibition of autophagy appears to reduce the protein levels of pEGFR and PD-L1. Moreover, the reduction of RKO cell viability was identified through western blot. The cells treated with 20μΜ of HCQ alone or in combination with 1μΜ of E (C or P), 0,5 μΜ I (PE, NI, IPI) for 24 hours. Treatment with HCQ, HCQ+C, HCQ+IPI, HCQ+C+NI, HCQ+C+PE, HCQ+C+IPI induces pEGFR (1.2 to 2.4 folds). HCQ+P, HCQ+NI, HCQ+P+NI, HCQ+P+PE, HCQ+P+IPI reduces pEGFR (0.2 to 0.8 folds). Autophagy inhibition is confirmed through the protein levels of p62 (1.1 to 2.8 folds) and increasing ratio of LC3II/LC3I (6.6 to 9.8 folds). Co-treatment of colo-205 with HCQ + E + I trigger apoptosis as it was identified through cleavage of caspase-3 and PARP **(Fig 2C).** The entire combinatorial scheme with HCQ and the main effect after 24 hours are presented in Table 1.

The differential biological effect of BRAF^V00E^ versus mtKRAS oncogenes has been the topic of several studies. To determine the role of KRAS in triple inhibition (A+E+I), another MSI-H colorectal cancer cell line, HCT116, was used. Using the MTT-viability assay, the response of HCT116 cell line were measured after treatments with 1μΜ of E (C or P), 0,5 μΜ I (PE, NI, IPI), 1μΜ of the specific MEK inhibitor PD-0325901, and 5mM autophagy inhibitor (A) 3-Methyadenine (3-MA). The entire combinatorial scheme and the main effect on cell viability after 72 hours are presented in Table 2.

**Table 2:** The combination of E, I, PD and A and the main effect on HCT116 CRC cell line.

HCT116	treatment	viabillity	autophagy	apoptosis	HCT116	treatment	viabillity	autophagy	apoptosis
E	C	NC	no	no	PD	PD	NC	PR	no
	P	NC	no	no	PD+E	PD+C	NC	no	no
I	NI	NC	no	no		PD+P	NC	no	no
	PE	NC	no	no	PD+I	PD+NI	NC	no	no
	IPI	NC	no	no		PD+PE	NC	no	no
E+I	C+NI	NC	no	no		PD+IPI	NC	PR	no
	C+PE	NC	no	no	A	3-MA	NC	IN	no
	C+IPI	NC	no	no	A+PD	3-MA+PD	NC	IN	no
	P+NI	NC	no	no	A+PD+E	3-MA+PD+C0	NC	IN	no
	P+PE	NC	no	no		3-MA+PD+P	NC	IN	no
	P+IPI	NC	no	no	A+PD+I	3-MA+PD+NI	NC	IN	no
A	3-MA	NC	IN	no		3-MA+PD+PE	NC	IN	no
A+E	3-MA+C	NC	IN	no		3-MA+PD+IPI	NC	IN	no
	3-MA+P	NC	IN	no					
A+I	3-MA+NI	NC	IN	no					
	3-MA+PE	NC	IN	no					
	3-MA+IPI	NC	IN	no					
A+E+I	3-MA+C+NI	NC	IN	no					
	3-MA+C+PE	NC	IN	no					
	3-MA+C+IPI	NC	IN	no					
	3-MA+P+NI	NC	IN	no					
	3-MA+P+PE	NC	IN	no					
	3-MA+P+IPI	NC	IN	no					

NC: not changed; IN: Inhibition; PR: present; +: <20% protein vs control; ++: <40%

**Fig 3:**
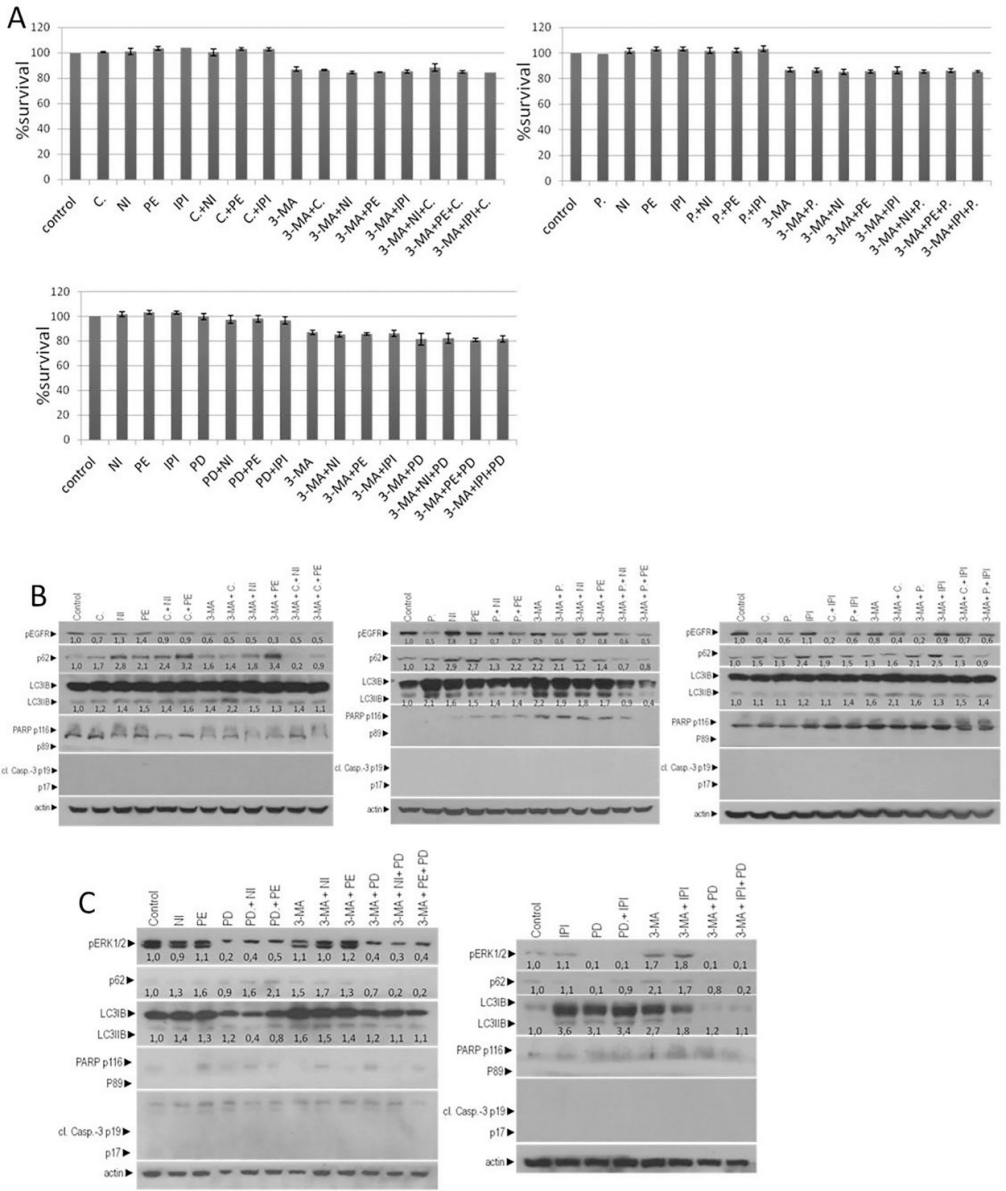
Triple inhibition does not affect the mutant KRAS/MSI-H CRC cell line. (**A**) MTT cell viability assay of HCT116 cell after 72 hours exposures of cells in constant dose of 1μΜ E, 0,5μΜ I, 1μΜ PD and in combination of E+I, A+E, A+I, A+E+I A+PD and A+PD+I. (**B**) The protein levels of p-EGFR, p-ERKs, LC3 and p62 are measured with Western blot analysis 24 hours after the exposure of HCT116 cells with a constant dose of 1μΜ E, 0,5μΜ I, and in combination of E+I, A+E, A+I, A+E+I. The protein levels of PARP and cl. Caspase-3 are measured for the identification of apoptosis. (**C**)Western blot analysis 24 hours after the exposure of HCT116 alone or in combination with a constant dose of 0,5μΜ I, 1μΜ PD, 5mM 3-MA and in combination of PD+I, A+I, A+PD, A+PD+I. The protein levels of apoptotic cell death were identified by antibody against PARP and caspase-3. The protein levels of p-ERKs, LC3 and p62 were measured by specific antibodies. The quantification of LC3 reflects the ratio of LC3II/LC3I in comparison with control in each sample separately. Protein levels were normalized against actin.

Due to inhibition of autophagy in double combination of A+E, A+PD and A+I, cell viability was not significantly changed; it was reduced up to 13,5% (A+P), 13,6% (A+C), 15,6% (A+NI), 15,2% (A+PE), 14,9% (A+IPI) and 19,5 (A+PD) after 72 hours **(Fig 3A).** Further inhibition of cell growth in triple inhibition of A+E+I and A+PD+I, was not observed. The total cell viability reduction in comparison with control was around 14,9–19,4% after 72 hours **(Fig 3A).**

Furthermore, CRC cell line HCT116, was exposed alone to 1μΜ of E (C or P), 0,5μΜ I (PE, NI, IPI), 1μΜ PD-0325901 and 5mM autophagy inhibitor (A) 3-Methyadenine (3-MA). The entire combinatorial scheme and the main effect after 24 hours are presented in Table 2 Treatment with C, P, C+NI, C+PE, C+IPI, P+NI, P+PE, P+IPI reduced pEGFR (0.2 to 0.7 fold). In contrast, treatment with NI, PE and IPI increased pEGFR (1.1 to 1.2 folds). In almost all treatment points, the ratio of LC3II/LC3I (in comparison with control) and p62 were increased (1.1 to 6.0 folds for LC3II/LC3I and 1.1 to 5.0 folds for p62). Treatment with MEK inhibitor (PD) reduced the protein levels of p62 (0.1–0.9 folds) as it was identified through western blot analysis. Treatment with PD+NI and PD+PE the ratio of LC3II/LC3I was decreased 0.4 to 0.8 folds and p62 was increased (1.8 to 2.6 folds). Furthermore, the protein levels of pERK1/2 were increased after treatment with IPI, A, A+PE, A+IPI (1.1 to 2.3 folds) and not significant change after treatment with NI or PE **(Fig 3B and 3C).** After inhibition of MEK (PD, PD+NI, PD+PE, PD+IPI, A+PD, A+PD+NI, A+PD+PE, A+PD+IPI) the protein levels of pERK1/2 were decreased 0.1 to 0.4. Due to inhibition of autophagy (A+E, A+I and A+E+I) the protein levels of pEGFR decreased around 0.2 to 0.9 folds as compared with untreated cells.

Upon inhibition of autophagy with 3-MA (A+E, A+I, A+PD, A+PD+I and A+E+I) increasing levels of LC3II/ LC3I ration was observed (1.1 to 11.6 folds). The protein levels of p62 were increased (1.1 to 6.5 folds) upon longer exposure to 3-MA (A+E, A+I, and A+E+I). Exception in this observation was the triple inhibition A+C+IPI which decreased the protein levels of p62 (0.9 folds). Moreover, the co-treatment of HCT11 cell lines with 3-MA and MEK inhibitor resulted in reduction of p62 (0.2 to 0.7 folds) in A+PD, A+PD+NI, A+PD+PE, A+PD+IPI treatment points **(Fig 3B and 3C).** In HCT116 cell line, these data confirmed that autophagy inhibition with 3-MA resulted in accumulation of LC3. Furthermore, treatment with 5mM 3-MA or MEK inhibition did not trigger apoptotic cell death in double and triple inhibition (A+E, A+I, A+PD, A+E+I and A+PD+I). The absence of apoptotic cell death in HCT116 cells after treatment with 3-MA was initially confirmed by the detection of un-cleaved PARP-1 and caspase-3 by Western blot analysis **(Fig 3B and 3C).**

### Triple inhibition of (A), (E) and PD reduces cell viability and induces apoptosis in MSS colorectal cancer cell lines

Unfortunately, in MSS CRC, which makes up the majority of the CRC tumors in clinical practice, no benefit has been shown with single PD-1/PDL-1 inhibition. In our study, we examined the effect of triple inhibition in MSS CRC cell lines, colo-205 and HT29. Colo-205 exposed alone or in combination with 1μΜ of E (C or P), 0,5 μΜ I (PE, NI, IPI), 1μΜ of the specific MEK inhibitor PD-0325901 and 5mM autophagy inhibitor (A) 3-Methyadenine (3-MA) on cell viability using the MTT-viability assay. The entire combinatorial scheme and the main effect on cell viability after 72 hours are presented in Table 3.

**Table 3:** The entire combinatorial scheme of E, I, PD and A and the and the main effect on colo-205 CRC cell line.

colo-205	treatment	viabillity	autophagy	apoptosis	colo-205	treatment	viabillity	autophagy	apoptosis
E	C	NC	NC	NC	PD	PD	+	PR	NC
	P	NC	PR	NC	PD+E	PD+C	+	PR	NC
I	NI	NC	NC	NC		PD+P	+	PR	NC
	PE	NC	PR	NC	PD+I	PD+NI	+	PR	NC
	IPI	NC	NC	NC		PD+PE	+	PR	NC
E+I	C+NI	NC	PR	NC		PD+IPI	+	PR	NC
	C+PE	NC	NC	NC	A	3-MA	+	IN	NC
	C+IPI	NC	NC	NC	A+PD	3-MA+PD	+	IN	PR
	P+NI	NC	NC	NC	A+PD+E	3-MA+PD+C	++	IN	PR
	P+PE	NC	NC	NC		3-MA+PD+P	++	IN	PR
	P+IPI	NC	NC	NC	A+PD+I	3-MA+PD+NI	++	IN	PR
A	3-MA	+	IN	NC		3-MA+PD+PE	++	IN	PR
A+E	3-MA+C	+	IN	NC		3-MA+PD+IPI	++	IN	PR
	3-MA+P	+	IN	NC	A	HCQ	+	IN	NC
A+I	3-MA+NI	+	IN	NC	A+E	HCQ+C	+	IN	NC
	3-MA+PE	+	IN	NC		HCQ+P	+	IN	NC
	3-MA+IPI	+	IN	NC	A+I	HCQ+NI	+	IN	NC
A+E+I	3-MA+C+NI	+	IN	NC		HCQ+PE	+	IN	NC
	3-MA+C+PE	+	IN	NC		HCQ+IPI	+	IN	NC
	3-MA+C+IPI	+	IN	NC	A+PD	HCQ+PD	+	IN	PR
	3-MA+P+NI	+	IN	NC	A+PD+E	HCQ+PD+C	++	IN	PR
	3-MA+P+PE	+	IN	NC		HCQ+PD+P	++	IN	PR
	3-MA+P+IPI	+	IN	NC	A+PD+I	HCQ+PD+NI	++	IN	PR
						HCQ+PD+PE	++	IN	PR
						HCQ+PD+IPI	++	IN	PR

NC: not changed; IN: Inhibition; PR: present; +: <20% protein vs control; ++: <40%

**Fig 4:**
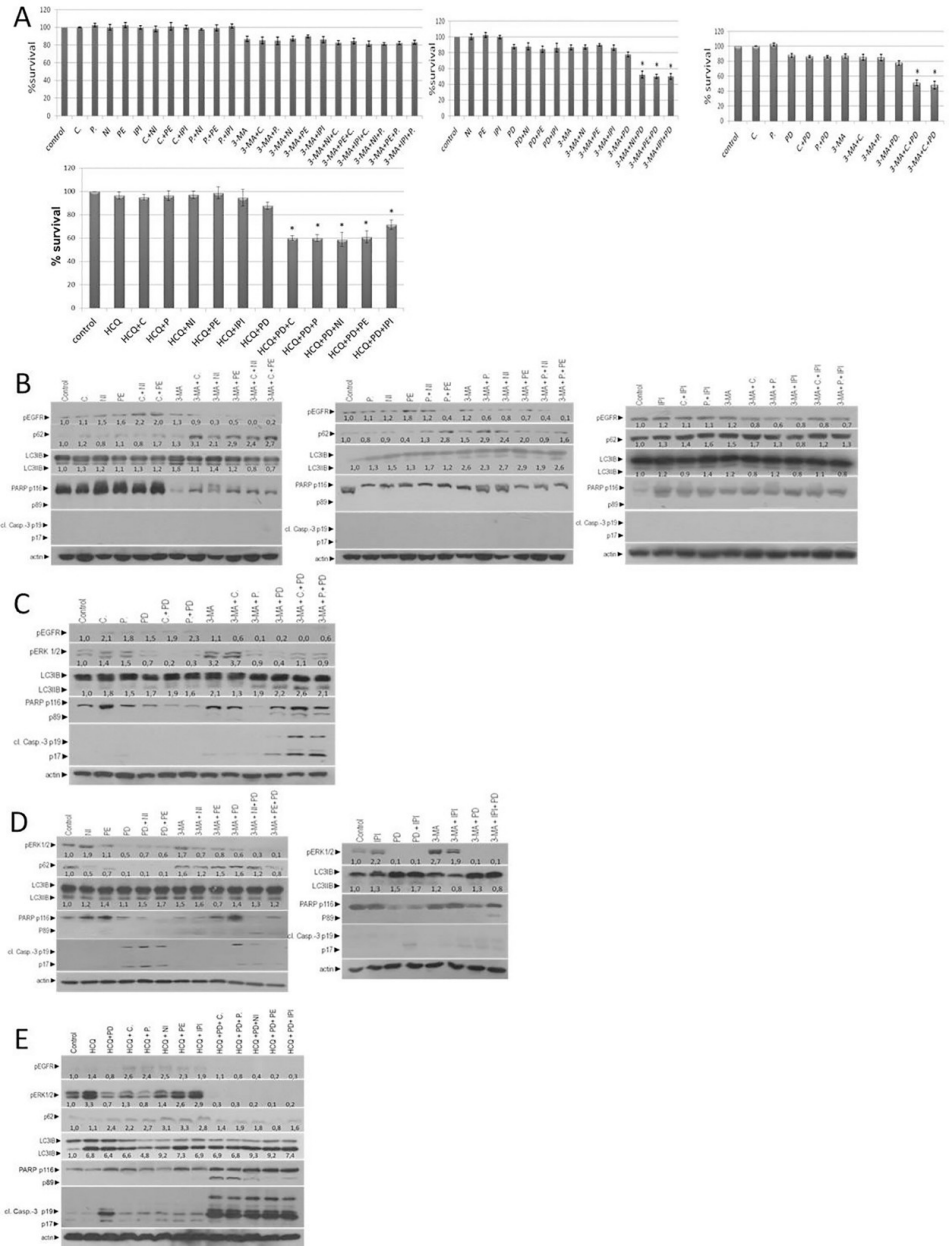
Autophagy and MEK inhibition enhances anti-EGFR treatment in MSS colon cancer cell line, colo-205. (**A**) Cell viability of colo-205 cell lines was measured, using the MTT assay, after treatment of cells for 72 hours with1μΜ of E, 0,5 μΜ I, 1μΜ of the specific MEK inhibitor PD-0325901, and 5mM of autophagy inhibitor (A) 3-Methyadenine (3-MA) or 20μΜ of Hydroxychloroquine (HCQ),alone or in combination of E+I, A+E, A+I, A+PD, A+E+I, A+PD+E and A+PD+I. (**B**) Western blot analysis after treatment of colo-205 for 24 hours with 1μΜ of E, 0,5 μΜ I and 5mM of autophagy inhibitor (A) 3-Methyadenine (3-MA), alone or in combination of E+I, A+E, A+I, A+E+I, A+PD+E and A+PD+I. the protein levels of p-EGFR, LCE3, p62, PARP and cl. Caspase 3 are detected through western blot analysis. The quantification of LC3 reflects the ratio of LC3II/LC3I in comparison with control in each sample separately. Protein levels were normalized against actin.(**C**) The protein levels of p-EGFR, p-ERKs, LCE3, p62, PARP and cl. Caspase 3 are measured with Western blot analysis 24 hours after the exposure of cells alone or in combination with 1μΜ of E, 1μΜ of PD-0325901 and 5mM of autophagy inhibitor (A) 3-Methyadenine (3-MA) alone or in combination of E+PD, A+E, A+PD, A+PD+E. (**D**) Western blot analysis after treatment of colo-205 for 24 hours with 0,5 μΜ I, 1μΜ of PD-0325901 and 5mM of autophagy inhibitor (A) 3-Methyadenine (3-MA) alone or in combination of I+PD, A+I, A+PD, A+PD+I. The detection of p-ERKs, LCE3, p62, PARP and cl. Caspase 3 is tested by specific antibodies against each protein. The quantification of LC3 reflects the ratio of LC3II/LC3I in comparison with control in each sample separately. Protein levels were normalized against actin. (**E**) Western blot analysis after treatment of colo-205 for 24 hours with 0,5 μΜ I, 1μΜ of PD-0325901 and 20μΜ of autophagy inhibitor (A) Hydroxychloroquine (HCQ), alone or in combination of A, A+PD, A+E, A+I, A+PD+E and A+PD+I. The detection of p-EGFR, p-ERKs, LCE3, p62, PARP and cl. Caspase 3 is tested by specific antibodies against each protein. The quantification of LC3 reflects the ratio of LC3II/LC3I in comparison with control in each sample separately. Protein levels were normalized against actin.

Double inhibition E+PD or E+I for 72 hours reduced cell viability 15% and 10,2–13,8% respectively compared with control. Due to inhibition of autophagy (A, A+E, A+I and A+PD) the cell viability was decreased 15%, 10,1–13,7% and 22,3%, respectively. Further inhibition of cell growth in triple inhibition of A+E+I was not observed (17,3–18,6%). In A+E+PD and A+I+PD a strong reduction in the total cell viability after 72 hours was detected (43–50%) **(Fig 4A).** Moreover, colo-205 CRC cell lines treated also with 20μΜ of a second autophagic flux inhibitor hydroxychloroquine (HCQ). All combinatorial schemes are shown in Table 3. Triple inhibition of A+E+I, using HCQ, reduce the cell viability in average of 55–60% after 72 hours **(Fig 4A).**

To further explore the mechanisms with which the triple inhibition affects the viability of MSS CRC cell colo-205, we assessed the protein levels of two specific apoptotic markers PARP-1 and caspase-3. Triple combinatorial scheme of A+PD+E and A+PD+I in colo-205 resulted in apoptotic cell death. The presence of apoptotic cell death was initially confirmed by the detection of PARP-1 cleavage and cleaved caspase-3 by western blot analysis after 24 hours **(Fig 4B–4D).** Treatment with E (C, P), I (NI, PE, IPI), E+I (C+NI, C+PE, C+IPI, P+NI, P+IPI), PD, E+PD (C+PD, P+PD) induced EGFR activation (1.1 to 4.9 folds), except in P+PE combination where pEGFR was decreased (0.2 folds). Moreover, after treatment with E (C, P), I (NI, PE, IPI), E+I (C+NI, C+PE, P+NI, P+PE, P+IPI), PD, E+PD (C+PD, P+PD) and PD+I (PD+NI, PD+PE, PD+IPI) autophagy was triggered as it was identified through the increasing levels of LC3II/I ratio (1.1 to 2.8 folds). In addition, treatment with P, NI, PE, C+NI, PD, PD+NI, PD+PE decreased the protein levels of p62 (0.0 to 0.4 folds) confirming the induction of autophagy in these treatment points. Treatment with C, C+PE, P+NI, P+PE, IPI, C+IPI, P+IPI resulted in increasing protein levels of p62 (1.1 to 2.7 folds) **(Fig 4B and 4C)**. Due to inhibition of autophagy (A+E, A+I, A+PD, A+E+I and A+E+PD), the protein levels of pEGFR were decreased around 0.7 to 0.1 folds as compared with untreated cells. Upon longer exposure to 3-MA, the accumulation of LC3 (1.2 to 1.6 folds) and p62 (1.2 to 9.3 folds), as it was identified through western blot, confirmed the autophagy inhibition **(Fig 4B and 4C).** Interestingly, co-treatment with autophagy and MEK inhibitor sensitized colo-205 cell line to E (C, P) and I (NI, PE, IPI), resulting in apoptotic cell death in triple combinatorial scheme (A+I+PD and A+E+PD). The presence of apoptotic cell death in colo-205 cells after inhibition of autophagy was initially confirmed by the detection of PARP-1 and caspase-3 cleavage by western blot analysis after 24 hours **(Fig 4B–4D).** All the combinatorial scheme and the main effect after 24 hours are presented in Table 3. Colo-205 CRC cell line, treated alone or in combination with 20μΜ of HCQ, 1μΜ of E (C or P), 0,5 μΜ I (PE, NI, IPI), 1μΜ of the specific MEK inhibitor PD-0325901for 24 hours. The combinatorial schemes are shown in Table 3. Treatment with HCQ, HCQ+C, HCQ+P, HCQ+NI, HCQ+PE induces pEGFR (1.4 to 2.5 folds). HCQ+PD, HCQ+PD+P, HCQ+PD+NI, HCQ+PD+PE, HCQ+PD+IPI reduce pEGFR (0.2 to 0.8 folds). The effect of MEK inhibitor on colo-205 CRC cell line is confirmed through the protein levels of pERK1/2 (0.1–0.7 folds). Autophagy inhibition is confirmed through the protein levels of p62 (1.1 to 3.3 folds) and increasing ratio of LC3II/LC3I (4.8 to 9.3 folds). Co-treatment of colo-205 with HCQ + PD + E or I trigger apoptosis as it was identified through cleavage of caspase-3 and PARP **(Fig 4E).**

**Fig 5:**
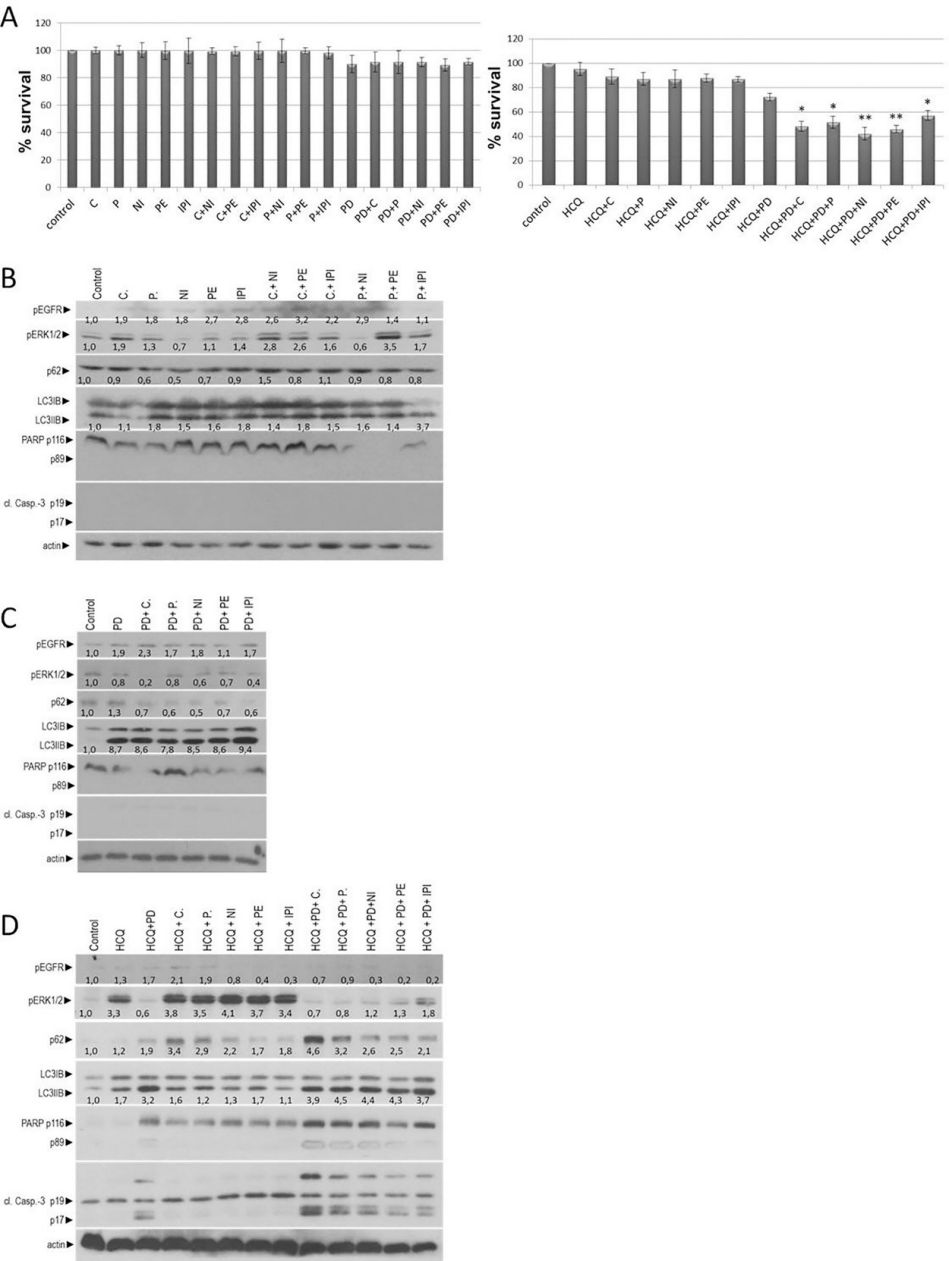
Inhibition of MEK and autophagy trigger apoptotic cell death in HT29, BRAV600E and MSS CRC cell line. (**A**) Cell viability of HT29 cell lines was measured, using the MTT assay, after treatment of cells for 72 hours with1μΜ of E, 0,5 μΜ I, 1μΜ of the specific MEK inhibitor PD-0325901, and (A) 20μΜ of Hydroxychloroquine (HCQ), alone or in combination of E+I, A+E, A+I, A+PD, A+E+I, A+PD+E and A+PD+I. (**B**) Western blot analysis after treatment of HT29 for 24 hours with 1μΜ of (E) and 0,5 μΜ (I) alone or in combination of E, I and E+I. The protein levels of p-EGFR, p-ERK1/2, LCE3, p62, PARP and cl. Caspase 3 are detected through western blot analysis. The quantification of LC3 reflects the ratio of LC3II/LC3I in comparison with control in each sample separately. Protein levels were normalized against actin. (**C**) The protein levels of p-EGFR, p-ERKs, LCE3, p62, PARP and cl. Caspase 3 are measured with Western blot analysis 24 hours after the exposure of cells alone or in combination with 1μΜ of (E), 0,5 μΜ (I) and 1μΜ of PD-0325901 alone or in combination of PD. E+PD and I+PD. (**D**) Western blot analysis after treatment of HT29 for 24 hours with 0,5 μΜ (I), 1μΜ of PD-0325901 and 20μΜ of autophagy inhibitor (A) Hydroxychloroquine (HCQ), alone or in combination of A, A+PD, A+E, A+I, A+PD+E and A+PD+I. The detection of p-EGFR, p-ERKs, LCE3, p62, PARP and cl. Caspase 3 is tested by specific antibodies against each protein. The quantification of LC3 reflects the ratio of LC3II/LC3I in comparison with control in each sample separately. Protein levels were normalized against actin.

The second BRAFV600E and MSS CRC cell line, HT29, exposed alone or in combination with 1μΜ of E (C or P), 0,5 μΜ I (PE, NI, IPI), 1μΜ of the specific MEK inhibitor PD-0325901 and 20μM autophagy inhibitor (A) HCQ for 24 hours. Co-treatment of HT29 cell line with HCQ+PD+E or I reduce cell viability around 40–57% after 24 hours **(Fig 5A).** The entire combinatorial scheme and the main effect are presented in Table 4.

**Table 4:** The entire combinatorial scheme of E, I, PD and A and the and the main effect on HT29 CRC cell line.

HT29	treatment	viabillity	autophagy	apoptosis
E	C	NC	PR	NC
	P	NC	PR	NC
I	NI	NC	PR	NC
	PE	NC	PR	NC
	IPI	NC	PR	NC
E+I	C+NI	NC	PR	NC
	C+PE	NC	PR	NC
	C+IPI	NC	PR	NC
	P+NI	NC	PR	NC
	P+PE	NC	PR	NC
	P+IPI	NC	PR	NC
PD	PD	+	PR	NC
PD+E	PD+C	+	PR	NC
	PD+P	+	PR	NC
PD+I	PD+NI	+	PR	NC
	PD+PE	+	PR	NC
	PD+IPI	+	PR	NC
A	HCQ	+	IN	NC
A+E	HCQ+C	+	IN	NC
	HCQ+P	+	IN	NC
A+I	HCQ+NI	+	IN	NC
	HCQ+PE	+	IN	NC
	HCQ+IPI	+	IN	NC
A+PD	HCQ+PD	+	IN	PR
A+PD+E	HCQ+PD+C	++	IN	PR
	HCQ+PD+P	++	IN	PR
A+PD+I	HCQ+PD+NI	++	IN	PR
	HCQ+PD+PE	++	IN	PR
	HCQ+PD+IPI	++	IN	PR

NC: not changed; IN: Inhibition; PR: present; +: <20% protein vs control; ++: <40%

Treatment with E (C, P), I (NI, PE, IPI), E+I (C+NI, C+PE, C+IPI, P+NI, P+IPI), induced EGFR activation (1.1 to 3.2 folds). Moreover, in the same combinatorial treatment p-ERK1/2 is increased (1.1 to 3.5 folds) except C and P+C where p-ERK1/2 is decreased (0.6 and 0.7 folds). The increasing ratio of LC3II/LC3I and the reduction of p62 identified the autophagy initiation after treatment with E, I and E+I. Inhibition of MEK with 1μΜ of PD decrease pEGFR (0.5 to 0.7 folds) and p-ERK1/2 (0.2 to 0.8 folds). Reduction of p-ERK1/2 leads to inhibition of autophagy as it was identified through the accumulation of LC3 and p62 (increasing ratio of LC3II/LC3I and p62). Apoptosis did not observe **(Fig 5B and 5C).**

After treatment of HT29 with autophagy inhibitor HCQ pEGFR is increased in HCQ, HCQ+PD, HCQ+C, HCQ+P (1.3 to 2.1 folds). Protein levels of pEGFR are decreased in HCQ+I, HCQ+PD+E or I (0.2 to 0.9 folds). Furthermore, inhibition of autophagy with HCQ increases the protein levels of p-ERK1/2 (3.3 to 4.1 folds). Inhibition of autophagy is confirmed through the increasing ratio of LC3II/LC3I and p62. Apoptotic cell death is observed after inhibition of autophagy with HCQ, in HCQ+PD+E or I, as it was identified through cleavage of capase-3 and PARP **(Fig 5D).**

These data demonstrate that dual inhibition of ERK and autophagy overcomes the resistance of colo-205 and HT29 to E and I.

### Autophagy-mediated resistance mechanism can be overcome by synergistic treatment of A+E+I for MSI-H cells and A+E+PD-0325901 or A+I+PD-0325901 in MSS cells in 3-D culture

The efficacy of triple inhibition on cell death properties were further analyzed, in CRC cell lines RKO, colo-205 and HT29, in conditions that mimic the real tumor microenvironment. Cells were grown in 3-dimensinal cultures and formed tumors in extracellular matrix. Nuclei were identified by DAPI staining (blue) and cleaved Caspase-3 (red) antibody under confocal microscopy.

**Fig 6:**
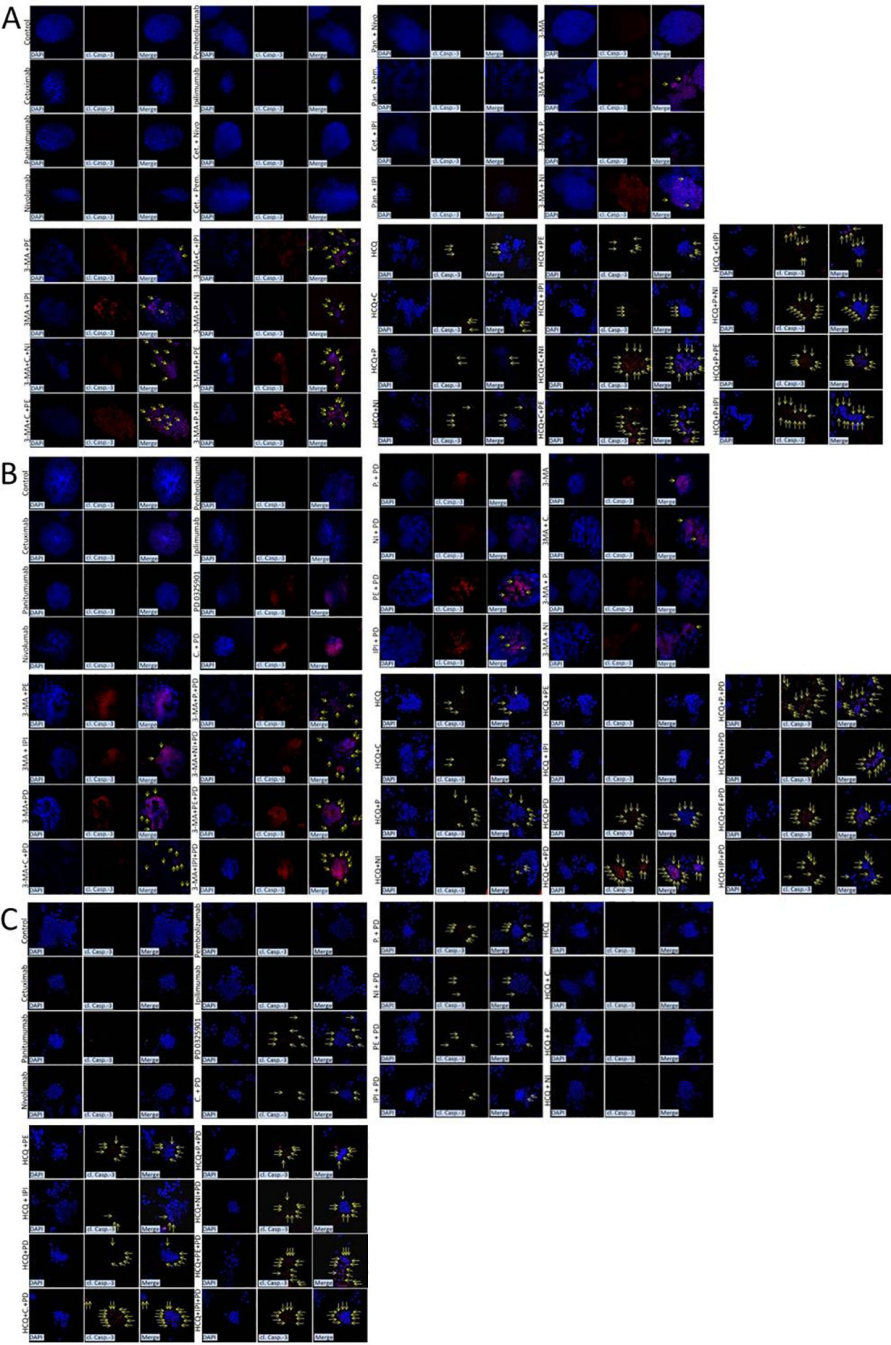
Autophagy inhibition reduces tumor mass in combination of immunotherapy and anti-EGFR treatment in colon cancer cell lines. Confocal microscope images of three-dimensional culture in RKO and Colo-205 cell lines.(**A**) RKO cells treated with 1μM anti-EGFR mAbs Cetuximab (C) or panitumumab (P), 5mM of the autophagic inhibitor 3-MA or 20μΜ of (HCQ), 0,5 μM checkpoint inhibitors nivolumab (N), Pembrolizumab (PE) or ipilimumab (IPI) for 48 hours alone or in 3-Methyladeninein combination with a constant dose of E, I and / or A. (**B**) Colo-205 cells treated with 1μM anti-EGFR mAbs Cetuximab (C) or panitumumab (P), 0,5μM of check point inhibitors nivolumab (NI), pembrolizumab (PE), ipilimumab (IPI), 5mM of the autophagic inhibitor 3-MA or 20μΜ of HCQ and 1μΜ MEK inhibitor PD-0325901 for 48 hours. Colo-205 were treated with [3-Methyladeninee (3-MA)] and in combination with a constant dose of E, I, PD, and / or A respectively for 48 hours. (**C**) HT29 cells treated 1μM anti-EGFR mAbs Cetuximab (C) or panitumumab (P), 0,5μM of check point inhibitors nivolumab (NI), pembrolizumab (PE), ipilimumab (IPI), 20μΜ of the autophagy inhibitor HCQ and 1μΜ MEK inhibitor PD-0325901 for 48 hours alone or in combination with a constant dose of E, I, PD and /or A for 48 hours. Nuclei were detected with DAPI (blue) and cleaved caspase-3 with the specific antibody (red). High concentration of cleaved caspase-3 and apoptotic nuclei are shown with yellow arrows.

In detail, RKO cells were treated with 1μΜ of E (C or P), 0,5 μΜ I (PE, NI, IPI) and 5mM autophagy inhibitor (A) 3-Methyadenine (3-MA) or 20μΜ of HCQ. Apoptotic cell death (apoptotic nuclei and cl. caspase-3) was not detected in monotherapy with anti-EGFR mAb (C, P) and checkpoint inhibitor (NI, PE, IPI) and in double inhibition E+I (C+NI, C+PE, C+IPI, P+NI, P+PE, P+IPI). In addition, the tumor mass did not significantly change. Inhibition of autophagy with both autophagy inhibitors (3-MA and HCQ) triggered apoptotic cell death as it was identified through cl. caspase-3 in both combinatorial schemes, A+E (A+C, A+P) and A+I (A+ NI, A+ PE, A+ IPI) but the tumor mass did not significantly change. As already demonstrated in previous figures, treatments involving autophagy inhibitors generally stabilized the expression of autophagic marker LC3 and triggered apoptotic cell death in RKO. The triple inhibition A+E+I (A+C+NI, A+C+PE, A+C+IPI, A+P+NI, A+P+PE, A+P+IPI) triggered a strong apoptotic cell death as it was observed through cleaved caspase-3 and apoptotic nuclei. Furthermore, the tumor mass was significantly reduced in 3D culture, where cells appeared apoptotic after treatment of tumors with 3-MA or HCQ followed by co-treatment of anti-EGFR mAbs (C, P) and checkpoint inhibitors (NI, PE, IPI) **(Fig 6A).** The entire combinatorial schemes are presented in Table 1.

In contrast with MSI-H cell line, the MSS cell lines colo-205 and HT29 was treated with 1μΜ of E (C or P), 0,5μΜ of I (NI, PE or IPI), 1 μΜ PD-0325901 and 5mM autophagy inhibitor (A) 3-Methyadenine (3-MA) or 20μΜ of HCQ. Apoptotic cell death (apoptotic nuclei and cl. caspase-3) was not detected in monotherapy with anti-EGFR mAbs (C, P) or with check point inhibitors (NI, PE, IPI). Specific MEK inhibitor PD-0325901 and double inhibition of E+PD (C+PD, P+PD) or I+PD (NI+PD, PE+PD, IPI+PD) slightly triggered apoptosis. As it was observed in MSI-H colon cell line, inhibition of autophagy also triggered apoptosis in MSS cell line as it was identified through cl. caspase-3 in both combinatorial schemes A+E (A+C, A+P), A+I (A+NI, A+PE, A+IPI) and A+ PD. In these treatments points, slightly reduction of the tumor mass was observed. The triple inhibition A+E+PD (A+C+PD, A+P+PD) and A+PD+I (A+PD+NI, A+PD+PE, A+PD+IPI) triggered strong apoptotic cell death as it was identified through cleaved caspase-3 and apoptotic nuclei and a significant reduction of tumor mass in 3D culture **(Fig 6B and 6C).**

These data confirmed that triple inhibition can trigger apoptotic cell death and reduced tumor mass in CRC cell lines bearing BRAF^V600E^.

### Induction of autophagy after treatment with ant-EGFR mAbs and checkpoint inhibitors

**Fig 7:**
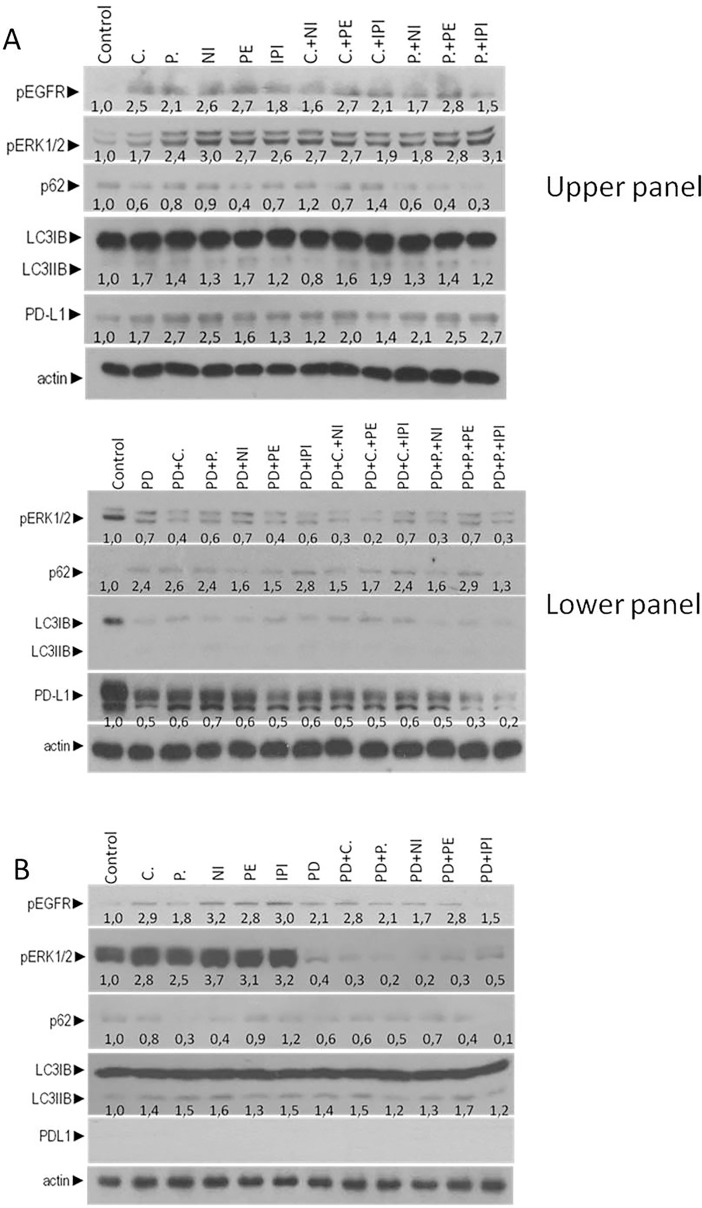
ERK-depended of autophagy induction in MSI-H and BRAF^V600E^ cell lines after treatment with checkpoint inhibitors or anti-EGFR mAbs. (**A**) Western blot analysis of protein levels of pEGFR, pERKs, LC3, p62, PD-L1 and actin after 24 hours treatment with 1μM anti-EGFR mAbs Cetuximab or panitumumab and 0,5 μM checkpoint inhibitors nivolumab, pembrolizumab or ipilimumab and the combination of E+I in mutant BRAF^V600E^ cell line RKO (upper panel) and in PD, PD+E (PD+C, PD+P), PD+I (PD+NI, PD+PE, PD+IPI) (lower panel). The quantification of LC3 reflects the ratio of LC3II/LC3I in comparison with control in each sample separately. (**B**) Western blot analysis of protein levels of pEGFR, pERKs, LC3, p62, PD-L1 and actin after treatment for 24 hours treatment with 1μM anti-EGFR mAbs Cetuximab or panitumumab and 0,5 μM checkpoint inhibitors nivolumab, pembrolizumab or ipilimumab, 1μM of MEK inhibitor PD 0325901 and in PD+E (PD+C, PD+P), PD+I (PD+NI, PD+PE, PD+IPI) in colo-205 cell line for 24 hours. The quantification of LC3 reflects the ratio of LC3II/LC3I in comparison with control in each sample separately.

We initially examined the effect of anti-EGFR mAbs Cetuximab (C), panitumumab (P) and checkpoint inhibitors nivolumab (N), pembrolizumab (PE), ipilimumab (IPI) on the autophagy and EGFR induction on colorectal cancer cell lines bearing BRAF^V600E^ (RKO and Colo-205), in order to have an initial indication on the potential regulation of autophagy by these agents. Both cell lines were exposed alone to 1μΜ of E (C or P) and 0,5 μΜ I (PE, NI, IPI) and in combination of E+I (C+NI, C+PE, C+IPI, P+NI, P+PE, P+IPI) for 24 hours. In all treatments the protein levels of pEGFR were increased 5.2 to 11.0 folds for RKO and 1.1 to 3.7 for colo-205 as compared with untreated cells. In RKO CRC cell line, treatment with C, P, PE, NI, IPI and the combination of C+PE, P+NI, P+PE, P+IPI induced autophagy as it measured through the increasing ratio of LC3II/I (1.2 to 2.2 folds) and the reduction of p62 (0.2 to 0.7 folds). In combination of C+NI p62 was increased (1.1 folds) and in C+IPI protein levels of p62 was decreased (0.2 folds). Furthermore, in RKO, the protein levels of pERKs and PD-L1 were increased 1.8 to 5.2 and 1.2 to 1.9 respectively **(Fig 7A, upper panel)**. In colo-205 cell lines treatment with C, P, NI, PE and IPI increased autophagy as it was identified through the increasing ratio of LC3II/I (1.1 and 1.4 folds) and the reduction of protein levels of p62 (0.2 to 0.8 folds) **(Fig 7B)**.

The hypothesis that anti-EGFR mAbs and checkpoint inhibitors regulate autophagy through the induction of ERKs was evaluated through the inhibition of MEK/ERK signalling pathway with the MEK inhibitor PD 0325901. RKO cell line was pre-treated with 1μΜ PD-0325901 and after was treated with 1μΜ Cetuximab (C), panitumumab (P) and 0,5μΜ checkpoint inhibitors nivolumab (N), pembrolizumab (PE), ipilimumab (IPI) alone and in combination for 24 hours. The protein levels of pERKs were decreased in all points (0.2 to 0.8 folds) and additionally the protein levels of PD-L1 were decreased to (0.1 to 0.8 folds) **(Fig 7A, lower panel).** Co-treatment of colo-205 with MEK inhibitor (PD 0325901**)** and C, P, NI, PE and IPI significantly decreased the protein levels of pERKs (0.0 to 0.2 folds). The ratio of LC3II/LC3I remained increased in comparison with untreated cells (1.1 to 1.5 folds) after treatment with PD **(Fig 7B).** These data identified the ERK-depended mechanism of autophagy induction and PD-L1 protein expression in MSI-H CRC cell line RKO after treatment with anti-EGFR mAbs and check point inhibitors. In contrary, in MSS CRC cell line colo-205, anti-EGFR mAbs and checkpoint inhibitors increased autophagy. These events would subsequently lead to the induction of autophagy independently of ERKs activation.

All these results indicate that autophagy represents a putative resistance mechanism of colon cancer cell lines bearing mutant BRAF^V600E^ to anti-EGFR mAbs (C, P) and checkpoint inhibitors (NI, PE, IPI) for MSI-H cell line RKO and MEK inhibitors for MSS cell line colo-205, since inhibition of autophagy by 3-MA could sensitize the cells to triple inhibition and apoptosis.

## Discussion

The present study supports the hypothesis that CRC tumors bearing BRAF^V600E^ and MSI-H or MSS phenotype are sensitive to autophagy inhibition. Consequently, the co-administration of anti-EGFR mAbs, checkpoint inhibitors and autophagy inhibitor can attenuate tumor growth. The mechanism which autophagy inhibition may be proven beneficial is strongly associated with the MSI status of CRC cells. To our knowledge, this is the first study to demonstrate that anti-EGFR mAbs and checkpoint inhibitors are responsible for the induction of the cytoprotective mechanism of autophagy in CRC cell lines bearing BRAF^V600E^.

Many studies have already identified the role of autophagy as a cytoprotective mechanism in several cancer types [28]. Furthermore, high autophagy levels in MSI tumors suggest a potential correlation between MSI status and autophagy as it is revealed through the subcellular localization LC3 with others specifics MSI markers [29,30]. In the present study a relevant correlation between BRAF^V600E^, MSI status and autophagy in CRC cell lines was found.

Several studies have so far highlighted the association between BRAF^V600E^ and autophagy in different cancer types, including CRC [12,31]. Autophagy is a key mechanism for tumor formation by promoting access to nutrients that are crucial to cancer metabolism, tumor growth and by inhibiting cellular death and increasing drug resistance [32,33]. However, due to the efficacy of EGFR-targeted mAbs in a small proportion of patients as well as the development of resistance, new treatment strategies have become the major focus in the field. Additionally, treatment with anti-EGFR mAbs results in deregulation of autophagy [34]. Most current findings support the notion that autophagy induced by anti-EGFR mAbs acts as a protective response in cancer cells [35,36].

Immunotherapy has been reportedly effective in colorectal cancers with high microsatellite instability. However, the specific cell types that respond to immune checkpoint therapy remain unclear [37]. Despite years of frustration, several studies are beginning to show encouraging results with immunotherapy in CRC [38]. In our study, we observed that, in RKO, a cell line which bearing BRAF^V600E^ and MSI-H phenotype, anti-EGFR mAbs and checkpoint inhibitors trigger autophagy through activation of MEK/EKR signalling pathway. It is well known that BRAF^V600E^ and MEK/ERK signaling pathway associated with the autophagy in mCRC. [12]. Furthermore, anti-EGFR mAbs and checkpoint inhibitors increase the protein levels of PD-L1 in RKO cell line. Several studies have already identified the present of high levels of PD-L1expression in MSI-H CRC tumors [39,40]. In our study, we observed an association between PD-L1 with MEK/ERK signaling pathway and autophagy. Inhibition of ERK or autophagy reduces the protein levels of PD-L1 in RKO. Several published data have shown that antibodies against PD1 or PD-L1 trigger autophagy in tumor cells, but not the vice versa effect [41].

**Fig 8:**
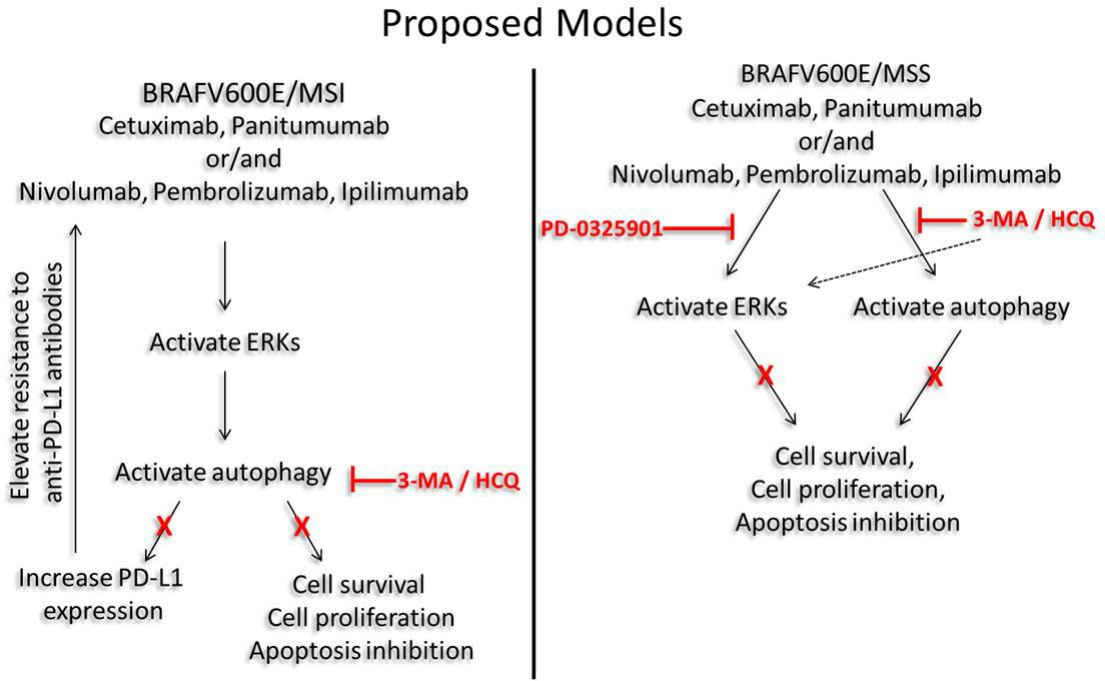
Proposed models of autophagy induction by anti-EGFR MoAbs and checkpoint inhibitors in CRC cell lines. Anti-EGFR mAbs (Cetuximab, panitumumab) and check point inhibitors (nivolumab, pembrolizumab, ipilimumab) trigger autophagy in MSI-H and MSS CRC cells in ERKs dependent and independent pathways, respectively. Moreover, inhibition of autophagy decreases the protein levels of PD-L1 in MSI-H cells. In MSS cells, the ERKs independent initiation of autophagy requires both autophagy and MEK inhibition. The triple inhibition A+E+I and A+PD+E/I initiate apoptotic cell death based on the microsatellite instability status.

From our experiments it is established that anti-EGFR mAbs and checkpoint inhibitors trigger autophagy in RKO, a resistance mechanism against anti-EGFR mAbs therapy. Moreover, autophagy induced by anti-EGFR mAbs acts as a protective response in cancer cells [34]. It is also well established that autophagy inhibition sensitizes cancer cells to apoptotic cell death. In a recent study in brain tumors bearing BRAF^V600E^, autophagy inhibition by Chloroquine sensitize cancer cells to BRAF inhibitor vemurafenib [42]. Another study, also found that the effect of pouranol was enhanced after co-treatment of pouranol with the autophagy inhibitor 3-Methyladenine (3-MA), results in apoptotic cell death of cancer cells [43]. A growing body of evidence through our experiments provides concrete data that inhibition of autophagy with 3-MA or HCQ followed by concomitant treatment with anti-EGFR mAbs and checkpoint inhibitors have a synergistic anti-tumor effect. This combinatorial scheme not only reduce cell viability but it is also sensitize RKO CRC cells (bearing BRA^V600E^ and MSI-H phenotype) to apoptosis, and tumor mass reduction **(Fig 8).**

As with RKO, in colo-205 and HT29, two CRC cell lines which also bearing BRAF^V600E^ but with MSS phenotype, anti-EGFR mAbs and checkpoint inhibitors trigger autophagy. Interestingly, colo-205 and HT29 appears to be more resistant to triple inhibition of A+E+I than RKO. This can be partially explained by a recent study which showed that the prognosis of MSS CRC tumors is strongly associated with CpG island methylator phenotype (CIMP) and BRAF^V600E^. According to this study, CRC patients with co-existence of mtBRAF and CIMP within MSS tumors have worse survival rates [44]. Colo-205 and HT29 are characterized by a high DNA methylation status of four CIMP-defining markers as it was revealed by the characterization of several CRC cell lines [45]. Moreover, we identified that autophagy which is triggered by anti-EGFR mAbs and checkpoint inhibitors is not associated with activation of ERKs, since inhibition of ERKs with specific MEK inhibitor did not affect the initiation of autophagy. Furthermore, both autophagy inhibitors 3-MA and HCQ seem to activate ERKs. These data suggest that in MSS cell lines with high CIMP status, colo-205 and HT29, an alternative therapeutic approach will be required. In preclinical models, specific inhibition of MEK leads to up-regulation MHC-I in tumors, enhances intratumoural T-cell infiltration and anti-PD-L1 mAbs efficacy [46]. In melanoma, colorectal, and breast cancer models, MEK inhibition upregulates IFN-γ mediated HLA molecule and PD-L1 expression [27,28]. In all of these models, MEK inhibition and PD-1 inhibition have been proven synergistic [38]. Therefore, the successful targeting of MSS CRC cell lines appears to be through inhibition of MEK. MEK and autophagy inhibition combined with anti-EGFR mABs or checkpoint inhibitors are being rigorously tested. Thus, MEK/ERK signaling pathway and autophagy regulation have a key role for improving the efficacy of anti-EGFR mAbs in mCRC with MSS phenotype [35]. Co-inhibition of MEK and autophagy sensitize MSS CRC cell lines, colo-205 and HT29, to anti-EGFR mAbs or checkpoint inhibitors. We demonstrate that triple inhibition (A+PD+E or A+PD+I) can overcome the resistance of MSS cell lines against anti-EGFR mAbs and checkpoint inhibitors and lead the cells to apoptotic cell death **(Fig 8).**

Approximately 50% of patients with metastatic CRC have somatic mutation of RAS oncogene [35]. Several studies have identified that the presence of mutant RAS is associated with poorer overall survival and increased risk of relapse in mCRC [47]. Furthermore, mutant RAS predicts response to anti-EGFR mAbs in first-line and beyond settings in the treatment of metastatic CRC [35]. In our experiments, we have shown that anti-EGFR mAbs and checkpoint inhibitors alone or in combination do not trigger autophagy in mutant KRAS and MSI-H CRC cell line, HCT116. In all combinatorial schedules, autophagy is inhibited as it was identified through the accumulation of p62 and LC3II. Furthermore, autophagy and MEK inhibition alone or in combination with anti-EGFR mAbs or checkpoint inhibitors do not trigger apoptotic cell death in mtKRAS cell line. These poor responses to our combinatorial approach may be explained by the controversial role of KRAS in autophagy regulation. It is well known that cancer cell lines bearing mutant RAS have higher levels of basal autophagy. Down-regulation of the expression of these autophagic proteins impairs cell growth [48]. Several studies have also suggested that mutant RAS prevents the autophagosome formation. Moreover, RAS regulate the degradation of Beclin-1, an essential autophagic protein, through activation of protease calpain. The reversal of the effect of mutant RAS in degradation of Beclin-1 can promote autophagosome formation [49]. These accumulated data point out that autophagy may is not crucial for CRC cell lines bearing mutant KRAS and MSI-H phenotype. The role of autophagy in cancer progression for tumors bearing mutant KRAS is not well understood and remains unclear. Further experiments are required to identify the effect of anti-EGFR mAbs and checkpoint inhibitors in mutant RAS driving autophagy.

## Conclusion

In summary, our data suggest that anti-EGFR mAbs and checkpoint inhibitors initiate autophagy in BRAF^V600E^ CRC cells. The combinatorial approach which we propose is associated with MSI status of CRC cells. For MSI-H cells triple inhibition of A+E+I represent an efficient option. Furthermore, PD-L1 expression and autophagy appears to be associated with MEK/ERK signaling pathway in BRAF^V600E^ and MSI-H CRC cells. In MSS cells, a different approach with co-inhibition of MEK and autophagy is required (A+PD+E or A+PD+I). Both therapeutic alternatives seem to attenuate the anti-EGFR mAbs and checkpoint inhibitors resistance in CRC cells and lead to apoptotic cell death. Our findings indicate that autophagy is a main mediator in the development of resistance against anti-EGFR mAbs and checkpoint inhibitors in CRC cells. In addition, MSI status of CRC cells is identified as a crucial predictive marker for the optimal therapeutic approach in BRAF^V600E^ CRC patients.

## Supporting information

S1 Fig Confocal images of basic levels of autophagy in CRC cell lines. The autophagic vacuoles were detected with 0,1mM of MDC (light blue) via confocal microscopy, while phalloidin staining (red) was used for cytoskeleton detection.
(TIF)

S1 Table Mutation table of CRC cell lines. Mutations of major oncogenes and the microsatellite status of CRC cell lines are presented in this table.
(PDF)
