# Supplementary figures and images for: The role of autophagy in the treatment of BRAF mutant colorectal carcinomas differs based on microsatellite instability status
Source: PLoS One. 2018 Nov 14;13(11):e0207227. doi: 10.1371/journal.pone.0207227 (PMC6241137; doi:10.1371/journal.pone.0207227)

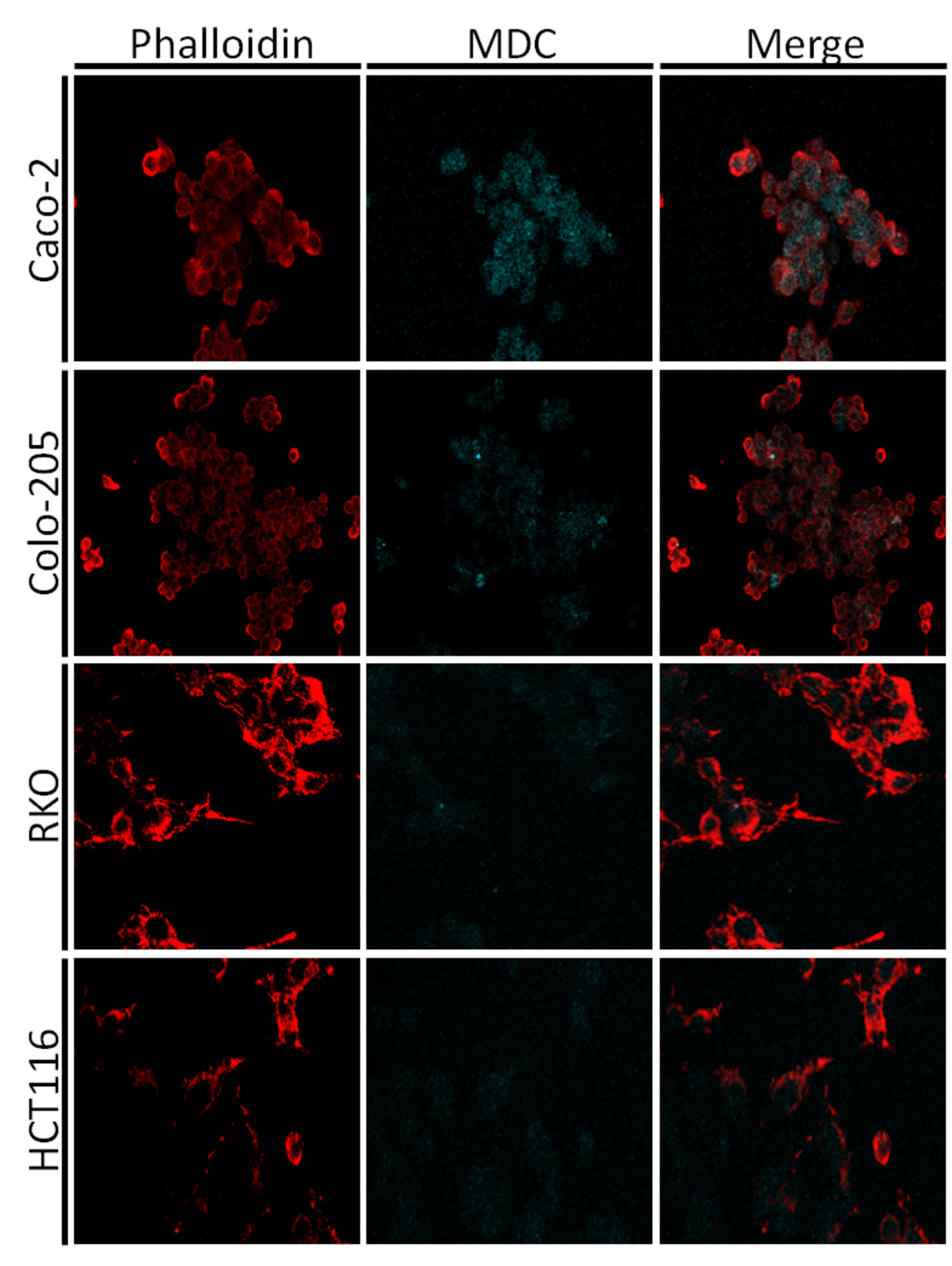

Supplement: S1 Fig — The autophagic vacuoles were detected with 0,1mM of MDC (light blue) via confocal microscopy, while phalloidin staining (red) was used for cytoskeleton detection. (TIF) [file pone.0207227.s001.tif]
