# Supplementary material for: The role of autophagy in the treatment of BRAF mutant colorectal carcinomas differs based on microsatellite instability status
Source: PLoS One. 2018 Nov 14;13(11):e0207227. doi: 10.1371/journal.pone.0207227 (PMC6241137; doi:10.1371/journal.pone.0207227)
Supplement: S1 Table — Mutations of major oncogenes and the microsatellite status of CRC cell lines are presented in this table. (PDF) [file pone.0207227.s002.pdf]

	Caco-2	RKO	colo-205	HT29	HCT116
BRAF	wt	$BRAF^{V600E}$	$BRAF^{V600E}$	$BRAF^{V600E}$	wt
KRAS	wt	wt	wt	wt	$KRAS^{G13D}$
PI3CA	wt	$PI3KCA^{H1047R}$		$PI3KCA^{P449T}$	$PI3KCA^{H1047R}$
EGFR	wt	wt	wt	wt	wt
Microsatellite status	MSS	MSI-H	MSS	MSS	MSI-H
